# Toward Precision Post-Stroke Rehabilitation Medicine: Integrating Molecular, Imaging, and Computational Biomarkers for Functional Outcome Prediction

**DOI:** 10.3390/jcm14228077

**Published:** 2025-11-14

**Authors:** Roxana Nartea, Simona Savulescu, Claudia Gabriela Potcovaru, Daniela Poenaru

**Affiliations:** 1 Department of Physical and Rehabilitation Medicine, Carol Davila University of Medicine and Pharmacy, 050451 Bucharest, Romania; simona.savulescu@umfcd.ro (S.S.); daniela.poenaru@umfcd.ro (D.P.); 2 National Institute for Rehabilitation, Physical Medicine and Balneoclimatology, 030079 Bucharest, Romania; 3 Elias University Emergency Hospital, 011461 Bucharest, Romania

**Keywords:** angiogenesis, bilirubin, corticospinal tract lesion load, electroencephalography, functional connectivity, ischemic stroke, machine learning, neurofilament light chain, neuroprotection, omentin-1, oxidative stress, thioredoxin, transcranial magnetic stimulation, diffusion tensor imaging

## Abstract

Ischemic stroke remains a leading cause of mortality and long-term disability worldwide, with prognosis influenced by heterogeneous biological and neuroanatomical factors. In the past decade, numerous possible biomarkers—molecular, imaging, and electrophysiological—have been investigated to improve outcome prediction and guide rehabilitation strategies and main objectives. Among them, neurofilament light chain (NFL), a cytoskeletal protein released during neuroaxonal injury, has become an effective marker of the severity of the neurological condition and the integrity of the neurons. Additional circulating biomarkers, including thioredoxin, netrin-1, omentin-1, bilirubin, and others, have been linked to oxidative stress, angiogenesis, neuroprotection, and regenerative processes. Meanwhile, innovations in electrophysiology (EEG and TMS-based predictions) and neuroimaging (diffusion tensor imaging, corticospinal tract lesion load, and functional connectivity) add some additional perspectives on the possibility for brain recovery. This work is a narrative synthesizing evidence from PubMed, Scopus, and Web of Science between 2015 and 2025, including both clinical and experimental studies addressing stroke biomarkers and outcome prediction. The review outlines a framework for the integration of multimodal biomarkers to support precision medicine and individualized rehabilitation in stroke.

## 1. Introduction

Ischemic stroke, caused by a disruption in cerebral blood flow, leads to (1) glial activation, (2) neuronal death, and (3) complex molecular cascades of excitotoxicity, oxidative stress, and inflammation. Even with significant advancements in neurorehabilitation and reperfusion therapy, the prognosis for motor and cognitive outcomes is still challenging [1,2]. Traditional clinical scales like the National Institutes of Health Stroke Scale (NIHSS) and modified Rankin Scale (mRS) give useful but limited information, as patients with comparable initial evaluations might have different recovery outcomes [3,4]. This indicates an increasing demand for objective biomarkers that can evaluate patients for focused therapies and reflect underlying pathophysiological processes.

**Figure 1:**
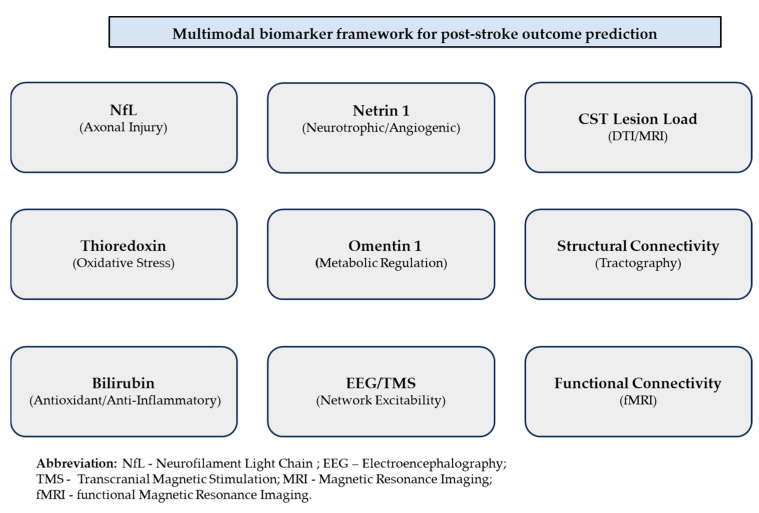
Multimodal biomarker framework for predicting post-stroke outcomes.

Over the last decade, technological innovations in proteomics, metabolomics, and neuroimaging have accelerated the discovery of novel biomarkers that capture neural damage and recovery potential more precisely. Among these biomarkers, due to its direct correlation with axonal damage and its detectable presence in blood and cerebrospinal fluid (CSF), the neurofilament light chain (NfL) has gained major interest [1]. At the same time, there have been links reported between stroke outcomes and metabolic regulators like omentin-1, neurotrophic and angiogenic modulators like netrin-1, and oxidative stress markers like thioredoxin [5,6]. Moreover, bilirubin, which was previously thought to be barely a byproduct of heme catabolism, is now acknowledged for having strong anti-inflammatory and antioxidant functions and becomes increasingly valuable as a predictive biomarker [7,8]. A visual schematic summarizing these molecular and neurophysiological biomarker domains is provided in Figure 1 to facilitate conceptual organization.

The figure summarizes representative molecular biomarkers (neurofilament light chain, thioredoxin, bilirubin, omentin-1, and netrin-1), neuroimaging markers (corticospinal tract lesion load, structural and functional connectivity), and electrophysiological measures (EEG and TMS-based excitability assessment). Together, these domains provide complementary insights into neural damage and recovery potential, supporting individualized rehabilitation planning.

This manuscript is a narrative review that synthesizes literature published between 2015 and 2025, identified through PubMed, Scopus, and Web of Science. Human clinical studies were prioritized, and preclinical studies were included where mechanistic clarification was necessary. Non-English language publications and studies focused exclusively on non-stroke neurological diseases were excluded. As this is a narrative synthesis, PRISMA methodological criteria were not applied. Keywords such as ischemic stroke, biomarkers, neurofilament light chain (NfL), bilirubin, thioredoxin, netrin-1, omentin-1, circular RNA, diffusion tensor imaging, EEG, TMS, motor recovery, and machine learning. Articles were included if they provided mechanistic insight, empirical data on prognostic performance, or demonstrated integration of multimodal predictors in human stroke cohorts. Additional references were drawn from key systematic reviews, meta-analyses, and cross-disciplinary studies in neurorehabilitation and computational neuroscience. 

## 2. Pathophysiologic Rationale for Biomarker Discovery in Stroke

To understand the promise and pitfalls of new biomarkers, it is helpful first to revisit the pathophysiology of ischemic stroke and recovery.

After an arterial occlusion, the ischemic core suffers rapid necrosis, while surrounding tissue in the penumbra may survive if reperfused promptly. Secondary injury cascades—oxidative stress, excitotoxicity, inflammation, blood–brain barrier disruption, apoptosis, and axonal degeneration—unfold over hours to days. In parallel, endogenous repair mechanisms (neuroplasticity, angiogenesis, synaptogenesis, and remyelination) are activated [1,3,4]. The net outcome is determined by a dynamic balance between injury and repair, modulated by collateral circulation, comorbidities (such as hypertension and diabetes), reperfusion success, and rehabilitation [1,2,5].

Biomarkers can, in principle, reflect one or more of those domains:

1. Structural injury (neuronal/axonal damage);
2. Oxidative stress/inflammation/glial injury;
3. Vascular injury, angiogenesis, endothelial health;
4. Network dysfunction/disconnection;
5. Repair/plasticity potential.

A reliable prognostic biomarker must fulfill a few criteria:

▪ Biologic plausibility (mechanistically linked to pathophysiology);
▪ Sufficient sensitivity and specificity for meaningful discrimination;
▪ Known temporal dynamics (e.g., how levels evolve from acute to chronic);
▪ Added predictive value beyond established clinical and imaging variables;
▪ Practicality (accessible, reproducible, cost-effective).

**Figure 2:**
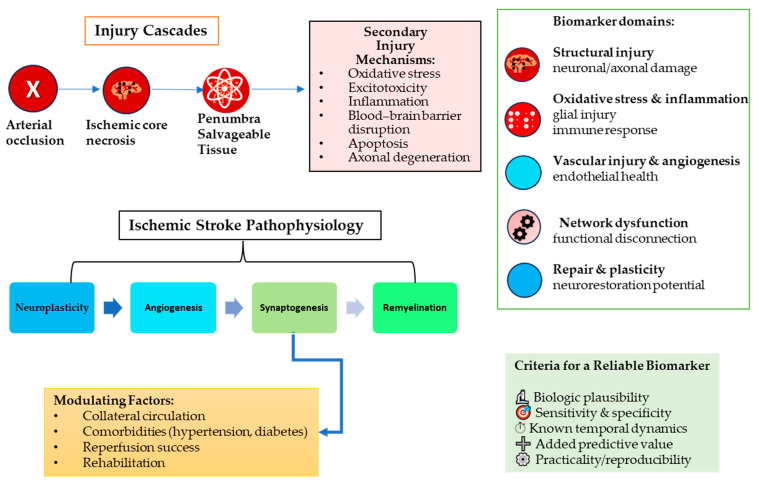
Pathophysiologic rationale for biomarker discovery in ischemic stroke.

With these in mind (see Figure 2), we review the main candidate biomarkers below.

## 3. Neuroaxonal Injury Biomarkers: Neurofilament Light Chain (NfL) and Peers

### 3.1. Biology of NfL and Its Relevance

Neurofilament proteins (light, medium, and heavy chains) are major structural components of the neuronal cytoskeleton, especially in axons. When axons are damaged (by ischemia, trauma, and degeneration), neurofilament proteins can be released into the extracellular space, then the cerebrospinal fluid, and ultimately into the blood, depending on the degree of blood–brain barrier disruption. The light chain (NfL) is particularly appealing as a biomarker because its molecular size is amenable to peripheral detection and it is relatively stable in biofluids [9,10,11,12].

In neurological diseases such as multiple sclerosis, Alzheimer’s disease, and traumatic brain injury, blood NfL (serum or plasma) has shown promise as a marker of ongoing axonal injury [9,10,13,14]. In ischemic stroke, NfL is thought to reflect the cumulative burden of neuroaxonal injury, encompassing both the primary infarct and secondary degeneration, and thus may correlate with infarct size, clinical severity, and subsequent recovery potential. Importantly, elevated NfL levels are not specific to central nervous system injury. In particular, patients with diabetes, especially those with diabetic polyneuropathy or subclinical peripheral nerve dysfunction, have been reported to exhibit higher circulating NfL concentrations [15,16,17]. Other neuropathic conditions, such as Guillain-Barré syndrome, similarly show increased NfL levels [13]. Because diabetes is itself a major risk factor for stroke, and peripheral nerve involvement may be clinically unrecognized, these comorbidities should be considered when interpreting NfL as a biomarker in stroke patients. Accounting for such systemic influences may improve the specificity of NfL measurements for central neuroaxonal injury and enhance prognostic models.

The advent of ultrasensitive immunoassays, particularly the single-molecule array (Simoa) platform, has allowed reliable detection of NfL in blood at concentrations previously measurable only in CSF. Because NfL release reflects neuroaxonal damage, it serves as a general marker of central nervous system injury and is increasingly recognized as a quantitative indicator of stroke severity and recovery potential [9,11,14].

### 3.2. Temporal Dynamics of NfL in Stroke

After an ischemic event, NfL concentrations in serum and plasma exhibit a distinct temporal profile (see Table 1) [9,14,15].

**Figure 3:**
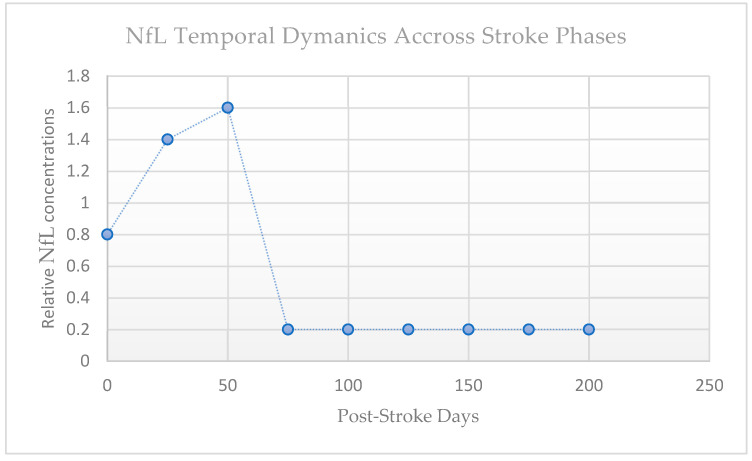
Temporal dynamics of NfL across stroke phases.

Early studies showed only a modest elevation in the acute phase (in the first few days), with levels peaking during the subacute period—approximately 2 to 4 weeks after onset [22]. This delayed rise likely reflects secondary axonal degeneration within the peri-infarct tissue and along white matter tracts, such as the corticospinal tract. In patients without recurrent damage, NfL concentrations then progressively decrease over the following months, reaching near-baseline values by 6 to 9 months (see Figure 3). White matter disconnection, lesion topography, and infarct volume are all correlated with the timing and size of the NfL peak [22].

This means that the biological window of assessment significantly determines interpretability. For instance, acute sampling (≤7 days) may underestimate overall axonal damage, whereas subacute values may more accurately represent the cumulative injury burden. Therefore, serial sampling provides light on the delayed degenerative and acute ischemic aspects of stroke pathophysiology. The kinetics of release, clearance, and diffusion into the systemic circulation are reflected in the latency of NfL in blood, which is one of the challenges. Three temporal moments were identified by a comprehensive review and meta-analysis that assessed the temporal pattern of blood NfL throughout stroke stages: acute (0–7 days), subacute (9–90 days), and chronic (>90 days) [9]. Remarkably, the peak in blood NfL typically occurs in the early subacute period (14–21 days) rather than immediately [9]. This underscores the need to carefully time sampling and interpret levels in context.

Recent studies further refine the temporal interpretation and prognostic relevance of circulating NfL after ischemic stroke. According to a 2019 study, serum levels of NfL rise in the acute phase and typically peak around one month, with higher concentrations strongly predicting poorer functional recovery at both 3 and 7 months, thereby reinforcing the value of subacute sampling [24]. Complementing these findings in a larger clinical context, it was reported that elevated plasma NfL independently predicted in-hospital mortality and long-term survival, indicating that NfL reflects both acute neurologic injury and systemic vulnerability [25,27]. A mechanistic confirmation that peripheral blood NfL changes closely parallel cerebrospinal fluid dynamics, validating serum measurement as a reliable proxy of central axonal injury, was observed [26]. More recently, a 2025 analysis demonstrated that admission NfL levels correlate with initial stroke severity and independently predict 90-day functional outcome, supporting their utility as an early stratification biomarker in routine clinical care [25]. Together, these studies strengthen the evidence that NfL is not only sensitive to the magnitude of neuroaxonal injury but also clinically meaningful across acute, subacute, and long-term recovery intervals.

In other words, NfL is not only a binary injury marker but may also offer gradation of injury burden and temporal staging. Because of these dynamics, comparing NfL levels across patients sampled at different timepoints can introduce confounding; thus, many studies stratify by sampling time or adjust for it.

### 3.3. Empirical Evidence of NfL in Ischemic Stroke Prognosis

Several clinical studies published between 2015 and 2025 suggest that elevated circulating NfL reflects both stroke severity and functional outcome. (see Table 1) In a 2018 prospective cohort study, it was reported that serum NfL strongly correlated with infarct size and 3-month modified Rankin Scale (mRS) scores [18]. In a 2019 study, Uphaus and colleagues confirmed that acute NfL levels predicted 90-day functional independence, outperforming certain conventional imaging markers [19]. Another study, published in 2022, observed that delta-NfL change from baseline to day 7 was linearly related to infarct volume and neurological improvement, suggesting that dynamic NfL monitoring adds prognostic precision [21]. In endovascular thrombectomy (EVT) cohorts, it was shown that higher plasma NfL concentrations are independently associated with poor outcomes despite successful recanalization, indicating persistent secondary injury [20]. A meta-analysis published in 2021, which includes results across several cohorts, concluded that plasma or serum NfL is a robust predictor of infarct burden and disability, though heterogeneity remains [28]. Other recent studies reinforced that both NfL concentration and its temporal trajectory can relate to early neurological deterioration and 3-month prognosis, supporting the biomarker’s clinical validity across populations [22,23].

In sum, the evidence supports that elevated serum NfL in the acute-to-subacute period is associated with worse functional outcomes, higher infarct burden, and increased mortality and/or recurrence of ischemic stroke.

Importantly, recent work supports the integration of NfL with neuroimaging-based predictors [13]. For example, corticospinal tract (CST) lesion load derived from diffusion-weighted MRI and diffusion tensor imaging (DTI) is a well-established structural marker of motor recovery potential [29]. When combined with serum NfL levels, predictive performance improves, particularly in differentiating patients with similar infarct volumes but differing degrees of secondary white matter degeneration. Likewise, models that incorporate NfL trajectories, lesion topology, and measures of network integrity (e.g., resting-state functional connectivity or tractography-based disconnectivity) demonstrate higher prognostic accuracy than either modality alone [13,15]. This suggests that NfL is most informative when interpreted alongside structural and network imaging metrics rather than used as a standalone indicator.

### 3.4. Advantages, Limitations, and Exceptions

The main benefit of NfL is its specificity for axonal damage, and also the possibility of non-invasive quantification using blood samples. Repeatedly measuring NfL can help track neuroaxonal integrity over time, unlike imaging techniques that require specific hardware. The biomarker integrates focal and diffuse injury processes and has the potential to complement neuroimaging or electrophysiological predictors, such as motor-evoked potentials or corticospinal tract lesion load, thereby improving prognostic modeling.

The NfL’s selectivity for axonal injury and its potential for minimally invasive assessment (using blood samples) are its main advantages. Neuroaxonal integrity can be evaluated over time with frequent measurements of serum NfL, unlike imaging methods that need specialized technology. By combining focal and diffuse damage processes, the biomarker may improve prognostic modeling by increasing neuroimaging or electrophysiological predictions, like motor-evoked potentials or corticospinal tract lesion extent.

Nonetheless, a few cautions are worth mentioning:

- NfL is not disease-specific. Dementia, traumatic brain damage, and demyelinating illnesses are among the various neurological disorders that have high levels, which could complicate interpretations specific to stroke.
- There is still a lack of test uniformity amongst laboratories. Absolute values can be affected by timing, platform calibration, and sample handling discrepancies.
- The third reason is that age-adjusted reference ranges are required because NfL concentrations scale with age and baseline neurodegeneration.
- In addition, when incorporating this biomarker into prognostic algorithms, the temporal heterogeneity of NfL kinetics highlights the necessity of specific sampling frames.

Despite these obstacles, current clinical studies suggest that serum and plasma NfL are among the most promising blood-based biomarkers for ischemic stroke prediction, especially when combined with imaging and clinical parameters in multiple-factor predictive frameworks.

Other neuroaxonal injury markers—including tau, UCH-L1, and glial fibrillary acidic protein (GFAP)—may capture complementary aspects of neuronal or glial pathology; however, their temporal dynamics and peripheral detectability are less consistent than NfL in ischemic stroke.

Current evidence suggests that integrated multimodal approaches combining NfL with white matter imaging and electrophysiology hold the greatest promise for precision outcome prediction [30,31,32,33] (See Table 2).

Additionally, assay harmonization remains a significant challenge. Although the single-molecule array (Simoa) platform offers high analytical sensitivity and reproducibility, values obtained using ELISA or other immunoassays are not directly interchangeable. Standardization efforts are ongoing to establish cross-platform reference calibrators and age-adjusted normative ranges.

It is important to note that circulating NfL levels may also be elevated in individuals with diabetes, especially in the presence of clinical or subclinical peripheral neuropathy, and in other peripheral neurodegenerative conditions. This may introduce confounding when interpreting NfL as a CNS-specific injury marker in stroke. Thus, integrating NfL with neuroimaging measures of corticospinal tract integrity and clinical phenotyping may improve specificity in prognostic models.

## 4. Oxidative Stress and Inflammation-Related Biomarkers

Due to several characteristics that promote the production of reactive species, including high oxygen consumption, high unsaturated fatty acid content, and elevated iron levels in certain brain regions, the brain is more susceptible to oxidative injury than other organs [34]. Additionally, the brain contains low levels and low activity of several antioxidant enzymes, such as glutathione peroxidase, catalase, and superoxide dismutase (SOD) [35]. The thioredoxin (Trx) system (Trx, NADPH, and thioredoxin reductase) and bilirubin represent two endogenous antioxidant systems of particular relevance in ischemic stroke [34,35,36].

### 4.1. Thioredoxin

Thioredoxin (Trx, often denoted Trx-1) is a small (~12 kDa) redox-active protein that maintains intracellular thiol homeostasis and limits oxidative damage by reducing disulfide bonds in target proteins [37]. In the central nervous system, Trx participates in mitochondrial protection, inhibition of apoptosis signal-regulating kinase 1 (ASK1), and suppression of NF-κB–mediated inflammatory cascades; experimental models show that augmenting Trx activity mitigates ischemic neuronal death and attenuates reperfusion injury [37,38].

However, interpretation depends on timing and physiological context. Moderate elevation may represent a protective compensatory response, whereas marked elevation may indicate overwhelming oxidative burden and cellular distress. Distinguishing these states requires time-resolved sampling rather than single measurements.

Clinically, several studies have investigated circulating Trx as an acute marker after ischemic stroke (see Table 3).

A prospective Chinese cohort reported that serum Trx concentrations were elevated in patients with first-ever acute ischemic stroke (AIS) compared with healthy controls and that higher Trx correlated with larger infarct size and some measures of early severity and prognosis [40]. The pattern is biologically plausible: ischemia–reperfusion generates reactive oxygen species (ROS) and oxidized proteins, which both induce Trx expression and increase extracellular leakage of Trx from stressed cells. However, the interpretation of elevated serum Trx is not unidirectional—it may reflect an adaptive compensatory antioxidant response (protective) or a marker of overwhelming oxidative burden (adverse), depending on timing and magnitude.

More recent experimental work demonstrates that exogenous Trx-related peptides or Tat-tagged Trx-like proteins reduce infarct volumes in rodent models, supporting the concept that Trx upregulation can be protective when it is functionally effective [38,39].

Despite mechanistic plausibility and promising single-center data, large multicenter validations are lacking. Key limitations include heterogeneity in assay methods, unclear reference intervals, confounding by systemic oxidative conditions (e.g., sepsis, cardiac ischemia), and sparse longitudinal sampling to define Trx kinetics post-stroke.

Thus, although mechanistically compelling, Trx requires standardized measurement protocols and multicenter validation before it can be widely used in clinical stroke care for prognostic purposes.

### 4.2. Bilirubin and Its Paradoxical Roles in Stroke

#### 4.2.1. Biologic Context

The bilirubin, produced during heme catabolism, is distributed primarily as conjugate (direct) and unconjugated (indirect), with the former being more frequently active than the latter. In addition to serving its traditional purpose as a liver function indicator, bilirubin is a potent endogenous antioxidant that modulates nitric oxide, inhibits NADPH oxidase, and scavenges peroxyl radicals by reducing inflammatory adhesion molecules and bioavailability [44,45,46]. As a result, bilirubin may provide vascular protection by reducing oxidative and inflammatory cascades thought to contribute to ischemic brain damage.

#### 4.2.2. The Connection Between Bilirubin and Stroke Risk

Epidemiological data assessing bilirubin as a risk factor for incident stroke are mixed. Mendelian randomization and large cohort analyses have not consistently supported a causal protective effect of higher bilirubin levels against stroke across populations. One MR study in Koreans found no overall causal association, whereas other MR or multivariable analyses suggest an inverse association for specific bilirubin fractions in some datasets [45,47]. Heterogeneity in genetic instruments, population structure, and bilirubin fraction examined (total vs. indirect vs. direct) likely explains discordant findings.

#### 4.2.3. Bilirubin and Ischemic Stroke Severity/Early Deterioration

Epidemiologic studies show inconsistent associations between bilirubin levels and incident stroke, likely reflecting population and fraction-specific differences [42,43]. In the acute stroke setting, lower admission bilirubin has been associated with a higher risk of early neurological deterioration (END), suggesting inadequate endogenous antioxidant reserve [41]. However, very high bilirubin levels, typically in the setting of hepatic dysfunction or hemolysis, correlate with worse outcomes, producing a nonlinear (U-shaped) relationship [48]. Recent cohort studies and meta-analyses suggest that moderate bilirubin levels within physiological ranges may be associated with a smaller infarct burden and better 3-month functional independence, whereas extremes on either end predict poorer outcomes [46,48]. Effects may vary by sex, liver function status, and metabolic comorbidities, underscoring the need for appropriately stratified interpretation.

Practical considerations:

- Liver dysfunction, hemolysis, and Gilbert syndrome;
- Fraction-specific reporting (total, direct, indirect);
- Value of serial sampling (admission, 24–72 h, 7–14 days).

#### 4.2.4. Comparative Perspective: Thioredoxin vs. Bilirubin (See Table 4)

In summary, Trx has stronger mechanistic specificity for oxidative injury, but it currently lacks diagnostic standardization. In contrast, bilirubin is readily measurable and clinically scalable, but it requires contextual interpretation due to its systemic influences.

**Table 4:** Thioredoxin (Trx) versus bilirubin.

Feature	Thioredoxin (Trx)	Bilirubin
Biological Role	Intracellular redox regulation and anti-apoptotic signaling	Systemic antioxidant reserve and vascular protection
Stroke Signal Meaning	Moderate elevation = adaptive; high elevation = oxidative overload	Moderate levels protective; very low or very high levels adverse
Assay Feasibility	Limited standardization; variable assay platforms	Routinely available, inexpensive, clinically standardized
Prognostic Strength (current evidence)	Mechanistically strong, but lacks multicenter validation	Clinical evidence moderate; strongest when interpreted with fractions and context

#### 4.2.5. Integrative Perspective: Bilirubin in the Context of Oxidative Stress Biomarkers

Viewed alongside other oxidative markers (e.g., Trx, glutathione peroxidase, and malondialdehyde), bilirubin has practical advantages: it is routinely measured, inexpensive, and widely available. However, unlike other stroke-specific markers (e.g., NfL for axonal injury), bilirubin reflects the systemic redox balance and the hepatic handling; thus, it must be interpreted with attention to comorbidities (liver disease, hemolysis, Gilbert syndrome). The most promising clinical approach is the use of multimarker panels that combine bilirubin (a general antioxidant reserve) with CNS-specific injury markers (NfL) and acute inflammatory mediators; such panels may help distinguish patients whose oxidative burden is outstripping their endogenous defenses from those mounting an effective protective response. These dynamics are better observed by serial measurements (admission, 24–72 h, and 7–14 days) than by single-timepoint assays. Standardized reporting of the bilirubin percentage (total, direct, and indirect), hepatic function correction, and prospective validation within multimodal prognostic models should be among the primary priorities for future studies.

In conclusion, Bilirubin is a promising predictive biomarker, in light of these studies, but there are several limitations:

- The dual nature (antioxidant vs. systemic stress signal) can determine some counterintuitive relationships.
- The timing of bilirubin measurement (admission versus serial) is important.
- Gender and comorbidity interactions can influence the association.
- Gilbert’s syndrome, hemolysis, and liver dysfunction need to be taken into account as modifiers.

In practice, bilirubin has the advantage of being generally available, affordable, and routinely evaluated in clinical labs, making it a low-hanging biomarker candidate for broader adoption once validated.

## 5. Vascular, Angiogenic, and Trophic Biomarkers in Ischemic Stroke

Beyond classical markers of neuroaxonal injury or oxidative stress, there is growing interest in biomarkers that capture endogenous repair, angiogenesis, and neurotrophic signaling after ischemic stroke. Such markers may indicate not only the extent of tissue injury but also the capacity for recovery and resilience. Among these, netrin-1, omentin-1, and circular RNAs (circRNAs) have emerged as promising, though still exploratory, candidates. See Table 5.

### 5.1. Netrin-1

Netrin-1 is a secreted laminin-related guidance cue originally characterized for its role in axonal pathfinding during neurodevelopment. In the mature brain, netrin-1 contributes to angiogenesis, blood–brain barrier maintenance, and modulation of inflammation and apoptosis through engagement of its receptors Deleted in Colorectal Cancer (DCC) and UNC5B [51,59,60]. In experimental stroke models, exogenous netrin-1 administration reduces infarct size, enhances microvascular density, and suppresses leukocyte infiltration [51].

Clinically, elevated serum netrin-1 concentrations during the acute phase have been associated with favorable functional outcomes. A case–control study of 180 ischemic stroke patients reported that individuals in the highest tertile of serum netrin-1 on admission had approximately threefold greater odds of achieving good functional outcomes (mRS ≤ 2 at 3 months) compared with those in the lowest tertile, even after adjusting for age and baseline NIHSS [49]. The biological rationale is that higher circulating netrin-1 may indicate an intact or more active reparative response, mitigating ischemic injury through pro-angiogenic and anti-apoptotic effects [50].

Evidence regarding stroke subtype differences is emerging but not yet definitive. Small single-center analyses suggest that the association between higher netrin-1 and a favorable outcome may be more pronounced in large-artery and cardioembolic stroke, where endothelial injury and angiogenic remodeling are central to tissue recovery, whereas findings in lacunar stroke (where pathology is more related to small-vessel lipohyalinosis) are less consistent. However, these subtype-specific interpretations remain tentative due to limited sample sizes and heterogeneous assay timing.

However, the field lacks large, longitudinal validation cohorts and standardized assays. Comparisons between studies are made more difficult by the variability of stroke subtypes and discrepancies in the time of blood analysis.

Thus, although netrin-1 is mechanistically well-supported, it remains an under-validated prognostic biomarker that requires prospective, subtype-stratified, multi-timepoint studies for further validation.

### 5.2. Omentin-1

Omentin-1 (also known as intelectin-1) is an adipokine primarily secreted by visceral adipose tissue and vascular endothelium. It exerts anti-inflammatory, insulin-sensitizing, and endothelial-protective effects, in part via activation of AMP-activated protein kinase (AMPK) and suppression of NF-κB signaling [54,61]. Given the well-established links between metabolic syndrome, vascular dysfunction, and stroke prognosis, omentin-1 has drawn attention as a potential systemic marker of metabolic and vascular resilience.

In a group of 150 patients with their first ischemic stroke, those in the lowest quartile of omentin-1 had significantly higher NIHSS scores on admission and worse 3-month outcomes than those in the highest quartile [52]. Multivariate analyses adjusting for age, BMI, and diabetes confirmed the independent association. Mechanistically, omentin-1 may counter oxidative stress, enhance endothelial nitric oxide synthase activity, and reduce atherosclerotic inflammation [52,53].

Nevertheless, the literature remains limited to small cohorts, and assay standardization (ELISA variants, fasting status) is inconsistent. As with netrin-1, additional longitudinal and interventional data are required before omentin-1 can be integrated into clinical prognostic models.

Current evidence suggests that circulating omentin-1 levels may reflect both baseline vascular-metabolic resilience and dynamic post-stroke metabolic adaptation. However, the relative contribution of these components remains unclear, and longitudinal studies are needed to distinguish pre-existing vascular health signatures from stroke-induced compensatory responses [61,62].

### 5.3. Circular RNAs and Other Noncoding RNAs

Noncoding RNAs (ncRNAs) have been described as important regulators and biomarkers in ischemic stroke, indicating post-transcriptional alterations in response to cerebral ischemia. Circular RNAs (circRNAs), a subclass of covalently closed, exonuclease-resistant RNA molecules, have garnered special interest due to their stability, tissue specificity, and regulatory complexity [57,63]. Mechanistic (preclinical) evidence: CircRNAs play a role in oxidative stress, inflammation, and apoptotic pathways during ischemic stroke, frequently operating as microRNA sponges or transcriptional modulators [64]. Experimental studies have shown that specific circRNAs, such as circDLGAP4, can protect against blood–brain barrier disruption and reduce infarct volume. This can suggest their neuroprotective potential [57].

Clinical association evidence: Elevated circulating levels of circDLGAP4 and hg38_circ_0008980 have been associated with stroke diagnosis, larger infarct volume, and worse short-term functional outcomes [56]. Meta-analyses indicate that dysregulated circRNA expression, particularly upregulation of pro-inflammatory circRNAs, correlates with poor recovery, underscoring their prognostic relevance [55]. Moreover, transcriptomic studies demonstrated that microRNAs and circRNAs form interactive regulatory networks influencing post-stroke inflammation, angiogenesis, and neuronal repair [58].

Beyond diagnostic applications, circRNAs may provide mechanistic insights into ischemic injury. For instance, several stroke-associated circRNAs regulate reactive oxygen species (ROS) signaling. This fact links RNA regulation to oxidative stress responses [64]. However, because detection platforms, normalization processes, and cohort sizes vary, translating this to clinical practice remains challenging.

Detection platform variability, normalization methods, and limited cohort sizes currently challenge clinical translation. Nevertheless, circRNAs represent a stable, mechanistically grounded, and promising class of biomarkers that may complement imaging and protein markers to improve stroke prognosis. Standardized measurement protocols and longitudinal, multicenter studies are needed to validate their predictive value.

## 6. Advanced Imaging and Electrophysiologic Biomarkers

Combining vascular and trophic biomarkers (netrin-1, omentin-1, and circRNAs) with injury markers (NfL) and oxidative stress indicators (thioredoxin and bilirubin) moves stroke assessment beyond lesion quantification to evaluating repair capacity and systemic resilience [65,66,67]. These markers reflect angiogenesis, metabolic regulation, and adaptive gene networks, complementing imaging and electrophysiologic measures of tract damage, connectivity, and cortical excitability. Together, molecular and structural metrics provide a multidimensional framework for predicting recovery, guiding individualized rehabilitation, and capturing the dynamic biology of stroke repair.

### 6.1. Tract Integrity Metrics: Corticospinal Tract (CST) and Lesion Load

One of the best indicators of post-stroke motor outcome is corticospinal tract (CST) integrity. It can now be measured using diffusion tensor imaging (DTI)-derived tractography. The CST’s lesion load predicts residual motor capacity and indicates the degree of axonal damage [68]. Fractional anisotropy (FA) asymmetry and tract-based spatial statistics provide quantitative indices correlating with Fugl–Meyer motor scores and long-term function [69,70].

In hyperacute and subacute phases, reduced CST FA predicts impaired voluntary activation, whereas preservation of tract continuity is associated with a favorable motor trajectory [70,71,72,73,74]. Large cohort studies confirm that lesion load explains substantial variance in recovery even after adjusting for infarct volume and age [75,76]. Combining CST injury metrics with clinical scales—such as the PREP2 algorithm—enhances early prognostic accuracy [77,78].

### 6.2. Functional Connectivity (Resting-State fMRI, Task fMRI)

The dynamic network interactions that facilitate motor recovery are clarified by functional magnetic resonance imaging (fMRI). Motor improvement is modulated by the resting-state connection between the contralesional homologs and the ipsilesional motor cortex, indicating compensatory plasticity [79]. Hyperconnectivity of default mode and salience networks in early phases may indicate maladaptive processes, whereas restoration of motor network segregation correlates with better outcomes [80].

Task-based fMRI demonstrates a shift from widespread bilateral activation acutely to focal, efficient ipsilesional recruitment during recovery—a biomarker of adaptive neuroplasticity. This reorganization is influenced by neuromodulatory interventions such as repetitive transcranial magnetic stimulation (rTMS), which normalizes motor cortex activation patterns [79,80].

Functional connectome analyses increasingly integrate graph theory measures, revealing that preserved network modularity and hub integrity predict responsiveness to therapy [81]. Together, these findings position functional MRI as both a prognostic and mechanistic biomarker of recovery potential.

Functional MRI (fMRI) evaluates the temporal evolution of motor network reorganization [79,80]:

- Acute phase: widespread bilateral cortical activation reflects recruitment of compensatory networks.
- Subacute phase: ipsilesional motor cortex gradually regains dominance, with progressive normalization of interhemispheric connectivity.
- Chronic phase: efficient, focal activation in ipsilesional networks correlates with better long-term motor recovery.

Resting-state fMRI identifies changes in connectivity between contralesional homologs and the ipsilesional motor cortex, indicating compensatory plasticity [79]. Task-based fMRI demonstrates adaptive shifts in recruitment patterns that can be enhanced with neuromodulatory interventions like repetitive TMS (rTMS) [80]. Functional connectome analyses, including graph-theory metrics, show that preserved network modularity and hub integrity predict therapy responsiveness [81].

### 6.3. Electroencephalography (EEG)/Brain Oscillations/Coherence Metrics

EEG offers a low-cost, bedside biomarker for the reconfiguration of cortical networks. During recovery, theta and alpha band synchronization are important indicators of interhemispheric balance, according to spectral and coherence investigations. One study published in 2020 found that the phase synchronization index (PSI) corresponds highly with upper limb motor progress, exceeding traditional EEG power measurements [82].

EEG-based brain–computer interface (BCI) paradigms that use motor imagery can improve sensorimotor feedback loops and accelerate recovery by reinforcing relevant oscillatory patterns [76]. Moreover, changes in beta coherence between motor cortices have been linked to functional gains, suggesting that EEG biomarkers may predict responsiveness to neurorehabilitative interventions [83].

EEG provides a low-cost, bedside assessment of cortical network reorganization [76,82,83]:

- Theta (4–7 Hz): reflects attentional control and interhemispheric coordination;
- Alpha (8–12 Hz): linked to sensorimotor inhibition and resting-state network stability;
- Beta (13–30 Hz): indicates motor planning and execution; increased coherence between motor cortices correlates with functional gains.

In general, alpha-band activity is considered to reflect functional network integrity and cortical idling dynamics, beta-band activity is associated with sensorimotor integration and movement preparation, while increased theta power may indicate disrupted network efficiency. These interpretations provide a physiological basis for linking EEG spectral features with post-stroke recovery trajectories [84].

EEG-based brain–computer interfaces (BCIs) leveraging motor imagery can reinforce beneficial oscillatory patterns and accelerate recovery [76].

### 6.4. Transcranial Magnetic Stimulation (TMS) + MEP-Based Prognostic Algorithms

Transcranial magnetic stimulation (TMS) offers a direct probe of corticospinal excitability. The presence or absence of motor evoked potentials (MEPs) serves as a robust biomarker of motor tract integrity. Integration of TMS-derived MEP data into the PREP2 algorithm allows accurate prediction of upper limb outcomes as early as two weeks post-stroke [77,78,85].

Beyond prognostication, repetitive TMS (rTMS) facilitates cortical reorganization by modulating the excitatory–inhibitory balance between hemispheres. High-frequency rTMS to the ipsilesional motor cortex or low-frequency stimulation to the contralesional hemisphere improves motor performance and enhances fMRI-measured connectivity [79]. These findings underscore TMS as both a predictive and therapeutic biomarker-driven tool in neurorehabilitation.

TMS directly probes corticospinal excitability. The presence or absence of motor-evoked potentials (MEPs) is a robust biomarker of motor tract integrity. Integration of MEP data into the PREP2 algorithm enables accurate early prediction of upper limb recovery [77,85].

Beyond prognostication, repetitive TMS (rTMS) can facilitate cortical reorganization by modulating interhemispheric excitatory-inhibitory balance. High-frequency stimulation to the ipsilesional motor cortex or low-frequency stimulation to the contralesional hemisphere improves motor performance and normalizes fMRI-measured connectivity [79,80]. Emphasizing clinical practicality, rTMS can guide individualized therapy, with timing and frequency optimized based on early biomarkers.

### 6.5. Comparative Overview of Imaging and Electrophysiologic Biomarkers

Advanced imaging and electrophysiologic biomarkers map the course of post-stroke healing. Structural parameters (CST integrity), functional network dynamics (fMRI), and cortical excitability markers (EEG, TMS) combine to form a multimodal framework for customized prognosis. See Table 6. Integrating various modalities, particularly through standardized algorithms such as PREP2, is a critical step for a specific rehabilitation individualized program, allowing for graded therapies that are linked with brain potential.

## 7. Integrated Prediction Models, Machine Learning, and Multimodal Biomarker Panels

Given the multifactorial nature of stroke recovery, single biomarkers seldom provide sufficient predictive accuracy to inform clinical decision-making. The heterogeneity of stroke—spanning lesion characteristics, systemic inflammation, neurophysiologic integrity, comorbidities (e.g., sleep disorders, vascular risk)—necessitates a multidimensional approach. Indeed, comorbid conditions such as chronic insomnia may interact bidirectionally with cerebrovascular risk and recovery mechanisms, potentially modifying biomarker trajectories and outcome predictions [96,97,98]. To meet these challenges, investigators increasingly combine clinical, imaging, electrophysiologic, molecular, and even lifestyle/circadian features into integrated prediction models. Advances in machine learning (ML) and AI now make it possible to synthesize these heterogeneous data streams into prognostic frameworks capable of refining individualized recovery trajectories and optimizing therapeutic strategies.

### 7.1. Machine Learning in Stroke Prognostication

In the last ten years, prognostic modeling for stroke has changed due to machine learning techniques. Conventional statistical techniques, such as logistic regression, are based on limited feature interactions and linear parameters. On the other hand, complicated nonlinear interactions between multimodal predictors and outcomes can be captured by supervised machine learning models, especially by ensemble methods like Extreme Gradient Boosting (XGBoost) and Gradient Boosting Machines (GBM). Research that applies gradient boosting to baseline CT and MRI data, in addition to demographic and clinical characteristics, significantly predicts 90-day modified Rankin Scale (mRS) results better than traditional regression models [74,99].

Beyond static predictors, deep learning models have been employed to analyze raw neuroimaging data. Deep learning models, including CNNs and transformer-based architectures, capture infarct evolution, biomarker trajectories, and dynamic clinical data. RNNs/LSTMs can be used for temporal sequences, but are included under this broader deep-learning category. Despite optimistic results, clinical translation is constrained by a number of factors. The majority of research uses small sample sizes and single-center datasets, which makes models more likely to overfit. There is little external validation across many populations, and interpretability—the so-called “black box” problem—remains a major barrier. Additionally, generalizability is restricted by missing data and variations in feature acquisition (such as assay standardization and MRI procedure variability). In order to suggest feature importance and promote clinical openness, explainable AI techniques like SHAP (SHapley Additive exPlanations) are becoming more utilised [77]. For example, SHAP analysis in a gradient boosting model may rank feature importance as CST lesion load > serum NfL > bilirubin, guiding clinicians toward the most informative, actionable markers [100,101]. Still, machine learning provides a strong foundation for combining high-dimensional, complex information into predictions that can be used in rehabilitation objectives.

### 7.2. Multimodal Biomarker Panels: Synergy and Redundancy

This multimodal integration reflects the complementary character of several biomarkers, including structural integrity (imaging), neuroaxonal damage (NfL), systemic oxidative stress (bilirubin), and network-level excitability or synchronization (EEG/MEP). However, the advantages of using biomarkers must be evaluated above the additional expense, complexity, and potential redundancy. Excessively linked features might impair model performance due to multicollinearity or overfitting [15,61]. Therefore, in order to preserve precision and generalizability, feature selection and dimensionality reduction (through methods such as LASSO regression, recursive feature elimination, or principal component analysis) are essential [71].

In addition to being accurate, clinically effective biomarker panels should be practical in everyday routines. This is why pragmatic models prioritize biomarkers that are reproducible, minimally invasive, and available during the acute or subacute phase of a stroke. Integrating a small but robust collection of markers (for example, imaging lesion load, serum NfL, bilirubin, and electrophysiologic measurements) might optimize the trade-off between reliability and practical implications.

### 7.3. Example of an Integrative Narrative Workflow

An example of an integrated prediction framework would look like this. (See Figure 2). To estimate the infarct core and penumbra, clinical information (age, baseline NIHSS, comorbidities) and imaging (CT/CTA) are obtained at admission. Diffusion MRI offers fractional anisotropy and CST lesion load measures as markers of white matter tract integrity in a matter of 24 to 48 h. Serum NfL and bilirubin levels, which indicate neuroaxonal damage and/or systemic oxidative stress, are obtained from blood samples taken between days three and seven. Cortical excitability and network connection are assessed concurrently using resting-state EEG or transcranial magnetic stimulation (TMS)-derived motor evoked potentials (MEPs).

**Figure 4:**
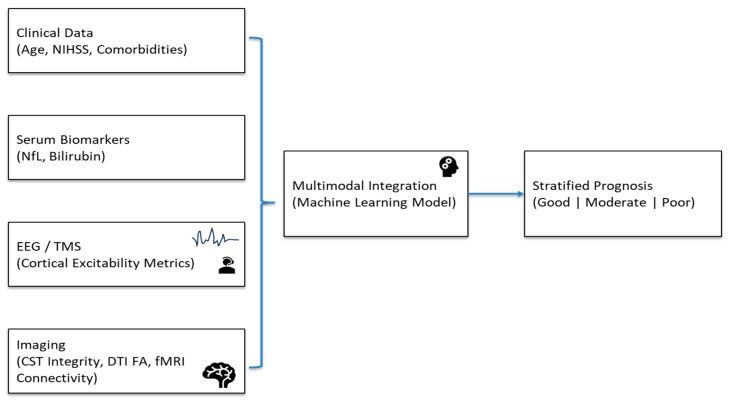
An example of an integrated prediction framework. Abbreviations: NIHSS—National Institutes of Health Stroke Scale; NfL—Neurofilament Light Chain; EEG—Electroencephalography; TMS—Transcranial Magnetic Stimulation; CST Integrity—Corticospinal Tract Integrity; DTI—Diffusion Tensor Imaging; FA—Fractional Anisotropy; fMRI—Functional Magnetic Resonance Imaging.

Figure 4 visualizes the data acquisition, integration, and prediction pipeline, showing how multimodal features are combined into an actionable recovery prediction framework for early rehabilitation planning. These multimodal features are then input into a trained gradient boosting or ensemble ML model to stratify patients into recovery likelihood categories (e.g., good, moderate, and poor). Figure 2 visually delineates the data acquisition, integration, and prediction pipeline, highlighting how biochemical, electrophysiologic, and structural indicators are combined into an actionable framework to guide early rehabilitation planning. Modular design allows flexibility if some data are missing.

### 7.4. Clinical Implications

Integrating machine learning-based, multimodal biomarker models into post-stroke rehabilitation care has the potential to substantially change prognosis and treatment decisions [65,66]. An early motor recovery potential prediction may help guide patient and family counseling, maximize resource allocation, and allow for customized rehabilitation intensity. Imaging-derived CST integrity combined with circulating NfL and bilirubin levels provides a multimodal measure of damage severity, systemic stress, and brain adaptation [67]. Furthermore, electrophysiologic metrics like MEP presence and EEG coherence convert this biology into bedside, real-time measures of brain activity [66].

By moving stroke prognostication from static scales toward precision neurorehabilitation, these frameworks support analytically interpretable, clinician-friendly AI systems and provide the basis for personalized, adaptive forecasting of recovery potential.

## 8. Discussion and Conclusions

In the last ten years, the prognostication of stroke outcomes has advanced from the use of static clinical scores to a multifaceted, systems-based approach that incorporates computational, molecular, imaging, and electrophysiologic biomarkers. This development represents a broader recognition of stroke as a complex, multifaceted process with systemic, vascular, and neural components. The combination of these modalities provides a more complete picture of post-ischemic pathophysiology, combining microscopic cellular injury with macroscopic network reconfiguration. It is now possible to integrate diverse data streams into predictive models that can anticipate unique recovery paths due to developments in computational techniques and artificial intelligence (AI), especially machine learning (ML).

### 8.1. Diagnostic vs. Prognostic Biomarkers

It is essential to distinguish between diagnostic biomarkers, which identify the presence or subtype of stroke, and prognostic biomarkers, which predict the recovery trajectory, functional outcome, or risk of complications. For example, acute serum NfL or bilirubin may assist in stratifying long-term motor recovery (prognostic), whereas imaging of perfusion deficits or diffusion restriction is primarily diagnostic. Recognizing this distinction helps guide the timing and selection of assessments and ensures appropriate integration into clinical decision-making workflows.

### 8.2. From Molecular Markers to Network-Level Dysfunction

Neurofilament light chain (NfL) has become one of the most reliable molecular indicators for axonal damage and long-term neurological sequelae. The infarct volume, baseline severity, and functional status after 90 days are all highly correlated with serum or plasma NfL levels, which usually peak 7–10 days after a stroke. At the same time, indicators of oxidative stress, such as bilirubin and thioredoxin, show systemic effects on neural recovery. While bilirubin plays a dual role—mild elevations may be neuroprotective, but high amounts predict worse outcomes—elevated thioredoxin indicates adaptive antioxidant action. The combination of NfL and redox-sensitive molecules captures both central (neuronal) and peripheral (systemic) features of ischemic damage.

### 8.3. Imaging and Electrophysiologic Correlates

Assessments of structural and network-level integrity are spatially detailed due to advanced neuroimaging. Especially for patients with severe impairment, diffusion MRI metrics (specifically fractional anisotropy (FA) and corticospinal tract (CST) lesion load) are among the most accurate indicators of motor recovery. In addition to focal injury, connectivity-based analyses show that neural network disconnection has a greater impact on functional outcomes than lesion volume alone. A strong interhemispheric connectivity, early after stroke, predicts favorable recovery, but excessive intra-hemispheric coupling may imply maladaptive response. These temporal insights are added by functional magnetic resonance imaging (fMRI). Dynamic measures of cortical excitability are provided by EEG and TMS, which improve imaging. The PREP2 algorithm, which integrates MEPs with clinical data, shows how electrophysiologic indicators can be successfully included in bedside prognostication.

### 8.4. Integration Through Machine Learning and Multimodal Modeling

Ensemble models like gradient boosting and XGBoost consistently surpass classical logistic regression at predicting 90-day outcomes. More sophisticated designs, such as recurrent neural networks (RNNs) and transformers, provide temporal modeling by combining changing biomarker trajectories with imaging results through time. Serum NfL levels, EEG or MEP features, and MRI-derived lesion metrics (all of which represent different but complementary biological aspects of neurological injury) are all included in the most effective multimodal workflows.

Explainable ML methods, such as SHAP, highlight feature contributions; for example, CST lesion load may rank highest, followed by NfL and bilirubin, guiding clinically actionable decisions. Clinical translation is limited by small samples, missing data, and lack of external validation, despite a promising AUROC (>0.80).

### 8.5. Ethical and Data Privacy Considerations

The use of AI and ML in stroke prediction raises important ethical and privacy issues. Patient data must be securely stored and processed in compliance with regulations (e.g., GDPR, HIPAA), particularly when integrating molecular, imaging, and electrophysiologic datasets. Transparency and interpretability are critical to maintain clinician and patient trust, while avoiding algorithmic bias that could exacerbate healthcare disparities. Consent, de-identification, and auditing of model outputs are recommended as best practices for ethical deployment of AI in stroke prognostication.

### 8.6. Conclusions

The integrative approach to post-stroke rehabilitation outcomes prognosis highlights the relationship between structural imaging, molecular markers, and network-level dynamics. Structural disconnection (imaging), axonal damage (NfL), systemic oxidative stress (bilirubin and thioredoxin), and cortical excitability (EEG/TMS) are all convergent axes to a single pathophysiological continuum. Personalized rehabilitation planning and trial grading will be made possible by future systems that integrate various modalities using verified, explicable machine learning algorithms.

Open access databases and large multicenter studies are essential for standardization and reproducibility. In the end, multimodal frameworks can help guide precise therapies and improve prognosis by customizing interventions according to the main biological pathways, whether they are structural, neurophysiologic, or inflammatory. The integration of molecular, imaging, and computational biomarkers thus marks a decisive step toward precision medicine in stroke recovery.

In conclusion, a pragmatic near-term roadmap might encompass the following strategies:

▪ Focus biomarker strategies on those with robust evidence and operational feasibility—namely, NfL and bilirubin;
▪ Use serial sampling (e.g., days 1, 7, 14) to capture temporal trajectories;
▪ Combine biomarker data with imaging (e.g., CST lesion load) and functional modalities (e.g., EEG, MEP) into a parsimonious multimodal prognostic model;
▪ Validate such models prospectively in multicenter cohorts with predefined sampling and outcome protocols;
▪ Ultimately, test whether biomarker-guided prognostication leads to improved patient outcomes (e.g., by tailoring rehabilitation, therapy allocation, or prognostic counseling).

## 9. Future Directions

To bridge the gap between discovery and clinical translation, stroke biomarkers research requires rigorous validation and standardization, temporal profiling, and data-driven customization.

1. Validation and Standardization

Larger multicenter investigations are required to determine reference ranges, temporal dynamics, and test repeatability. Standardized sampling techniques and analytical platforms will improve comparability and encourage clinical adoption.

2. Temporal Profile

Stroke rehabilitation is an ongoing process. In order to guide the best course of therapy and analyze rehabilitation, biomarkers including neurofilament light chain (NfL), thioredoxin, and bilirubin can be serially assessed during acute, subacute, and chronic phases. This can reveal new mechanisms of neuronal injury and repair.

3. Combination Panels

The accuracy of diagnosis and prognosis may be improved by composite panels that combine neuronal injury (NfL), oxidative stress (thioredoxin, bilirubin), and angiogenic markers (netrin-1, omentin-1). Such integrated rehabilitation techniques better reflect the multiple aspects of stroke pathogenesis.

4. Therapeutic Outcomes

Several biomarkers, like netrin-1 and omentin-1, may be therapeutic targets beyond prediction. After ischemic injury, altering these paths may encourage angiogenesis, neurorepair, and functional rehabilitation.

5. ML Customization

Individualized rehabilitation trajectories can be produced using machine-learning models that integrate biomarker, imaging, and clinical data, improving the intensity of rehabilitation and the distribution of resources.

Future progress will benefit from the adoption of open data standards, such as BIDS and FAIR principles, which enable reproducibility, cross-cohort harmonization, and the pooling of biomarker datasets. Multicenter consortia focused on assay standardization and longitudinal biomarker validation will be critical for clinical translation.

In conclusion, the future of post-stroke biomarker research depends on balancing computational accuracy with standardized validation to provide genuinely individualized and mechanistically informed post-stroke rehabilitation programs.

## Figures and Tables

**Table 1:** Temporal dynamics of NfL. Abbreviations: sNfL—serum/plasma neurofilament light; EVT—endovascular therapy; mRS—modified Rankin Scale; NIHSS—National Institutes of Health Stroke Scale; WMH—white matter hyperintensities; HD—hospital day; CV—cardiovascular; mo—month; yr—year.

Citation (Journal, Year)	Sample Size (Patients/Controls)	Assay Used (Platform/Kit Where Reported)	Sampling Timepoints (Relative to Stroke Onset/Admission)	Key Result
Steffen Dieth et al. 2018 [18]	196 (serial n = 89), 95; 30 controls	Simoa	↑ Admission → Day 7; 6-mo follow-up	sNfL ↑ post-stroke, peak ~Day 7; correlates with infarct volume; predicts 3-mo mRS
Uphaus T. et al. 2019 [19]	211	Simoa	Admission (<24 h), 1 yr	Admission sNfL correlates with NIHSS & WMH; predicts 90-day outcome & long-term CV events
Chen C.H. et al. 2021 [20]	60 EVT/14 controls	Simoa	Pre-EVT → Post → 24 h	NfL elevated vs. controls; higher NfL predicts 90-day death/disability; rises further by 24 h
Ahn J.W. et al. 2022 [21]	15/8 controls	Simoa	Admission, Day 7	NfL ↑ Day 1→7; Day 7 correlates with infarct volume; ΔNfL reflects infarct size
Holmegaard L. et al. 2024 [22]	316	Simoa HD-X	Acute → 3 mo → 7 yr	Acute & 3-mo sNfL reflect infarct size; non-linear decline (peak ~1 mo, 50% ↓ by 3 mo)
Xu C. et al. 2024 [23]	319	ELISA	Admission (<24 h).	Elevated sNfL predicts early deterioration & 3-mo poor outcome; adds to NIHSS/WMH
Sánchez J.D. et al. 2022 [9]	9 studies; ~4237	Mostly Simoa	Acute (0–7 d), Subacute (9–90 d), Chronic (>90 d).	Peak ~14–21 d; discriminates stroke vs. TIA; correlates with infarct size and outcomes
Pedersen A. et al. 2019 [24]	286 stroke/70 controls	Simoa	Acute (0–7 d), 3 months, 7 months	sNfL increased in acute phase, peaked ~1 month; higher levels predicted worse 3-month and 7-month functional outcomes.
Tony A.A. et al. 2025 [25]	120 acute stroke/60 controls	Simoa	Admission (≤72 h)	Admission sNfL correlated with NIHSS and infarct size; higher levels predicted poorer 90-day mRS; proposed use as routine severity marker.
Pujol-Calderón F. et al. 2019 [26]	30 stroke (paired serum + CSF)/18 controls	ELISA (serum and CSF)	Admission (24–48 h), day 7	NfL elevated in both serum and CSF; serum changes paralleled CSF dynamics; serum NfL reliably reflected central axonal injury.
Gendron F.T. et al. 2020 [27]	228 stroke	Simoa	Admission and serial during hospitalization	Plasma NfL predicted in-hospital mortality and long-term survival; prognostic value independent of age and NIHSS.

**Table 2:** NfL vs. other neuroaxonal/glial biomarkers [30,31,32,33].

Biomarker	Primary Source	Pathology Reflected	Peripheral Detectability	Temporal Profile	Key Limitations
NfL	Axons	Axonal injury	High (Simoa assays)	Delayed subacute peak (2–4 weeks)	Not disease-specific; age-dependent
Tau	Neuronal microtubules	Neuronal structural disruption	Variable; peaks early; less stable	Early acute rise (hours–days)	Short half-life; assay variability
UCH-L1	Neuronal cell bodies	Neuronal cell body damage	Moderate; better characterized in TBI	Early acute rise (hours–days)	Less validated in ischemic stroke
GFAP	Astrocytes	Astroglial activation/glial injury	Detectable early in the blood	Acute rise (hours–days)	More glial than axonal specificity

**Table 3:** Oxidative stress and inflammation-related biomarkers. Abbreviations: AIS—acute ischemic stroke; TRX—thioredoxin; NIHSS—National Institutes of Health Stroke Scale; mRS—modified Rankin Scale; TBil—total bilirubin; DBIL—direct bilirubin; END—early neurological deterioration; OR—odds ratio; aOR—adjusted odds ratio; CI—confidence interval; mo—months.

Study	Sample Size (Patients/Controls)	Assay (Platform/Method)	Main Effect Estimate (Reported)	Adjusted for (Covariates)
Qi A-Q et al. 2015 [39]	198 AIS vs. 75 controls	Solid-phase sandwich ELISA for serum TRX.	Elevated TRX (≥20.0 ng/mL) aOR 9.48 for poor short-term functional outcome (mRS at 3 mo). Also, TRX correlated with NIHSS (r = 0.476).	Adjusted for NIHSS and vascular risk factors and other predictors (multivariable logistic regression).
Wu MH et al. 2016 [40]	312 AIS	ELISA (solid-phase sandwich) for TRX.	TRX was higher in AIS (median 15.03 vs. 8.95 ng/mL); TRX was independently associated with stroke (adjusted OR 1.245 per unit; *p* < 0.0001) and with NIHSS (r ≈ 0.48).	Multivariable models adjusting for conventional vascular risk factors and potential confounders.
Oraby MI et al. 2020 [34]	100 subjects (50 AIS, 50 control)	ELISA/regionally reported assays.	The review concludes that TRX is often elevated in AIS and correlates with severity in several small cohorts; however, the results are heterogeneous.	Variable: most primary studies adjusted for age, NIHSS, and vascular risk factors.
Sheng X et al. 2021 [41]	291 AIS	Clinical chemistry on Immulite 2000 (Siemens)—serum total bilirubin (TBIL) measured.	Lowest TBil quartile (<9.8 μmol/L) associated with higher odds of END; after multivariable adjustment, low TBil remained independently associated with END (exact aORs reported in text). ~21.6% developed END.	Adjusted for demographics, vascular risk factors, baseline NIHSS, and other lab variables (multivariable logistic regression).
Ouyang Q et al. 2021 [42]	11,121 patients	Routine hospital laboratory bilirubin (TBIL, DBIL, IBIL) was measured and analyzed by quartiles.	Patients in the highest TBIL quartile had a higher risk of poor functional outcome (mRS 2–6) at 3 mo: OR 1.37 (95% CI 1.19–1.59) vs. the lowest quartile. J-shaped associations were observed.	Adjusted for age, sex, NIHSS, vascular risk factors, stroke subtype, concomitant labs, and other clinical confounders (extensive multivariable models).
Peng Q et al. 2022 [43]	718 AIS	Routine bilirubin fractions (TBIL/DBIL/IBIL) measured.	Different bilirubin fractions variably predicted outcomes after thrombolysis; DBIL sometimes had stronger associations with poor outcomes/END outcomes, or endpoints than TBIL.	Adjusted models included age, NIHSS, onset-to-treatment time, and comorbidities. (See paper for effect sizes.
Sun M et al. 2024 [44]	344 AIS	Clinical lab bilirubin (TBIL, DBIL, IBIL); tertiles used.	In women, the highest TBIL tertile vs. the lowest: adjusted OR 5.24 (95% CI 1.50–18.35) for END; DBIL independently associated with END (aOR per unit 1.72). No similar effect in men.	Adjusted for age, NIHSS, and other clinical confounders (multivariable logistic regression with sex-stratified analyses).
Song Y.J. et al. 2022 [8]	Meta-analysis pooling multiple studies (number and n vary)	Aggregated from routine clinical assays across studies.	Higher bilirubin associated with increased stroke severity (dose-response); authors caution heterogeneity & confounding.	Most included studies adjusted variably; meta-regression was used to explore heterogeneity.

**Table 5:** Evidence summary for vascular, angiogenic, and trophic biomarkers (netrin-1, omentin-1, and circular RNAs) in ischemic stroke. Abbreviations: AIS—acute ischemic stroke; ELISA—enzyme-linked immunosorbent assay; mRS—modified Rankin Scale; OR—odds ratio; CI—confidence interval; IMT—intima–media thickness (carotid); CRP—C-reactive protein; qRT-PCR—quantitative reverse transcription polymerase chain reaction; circRNA/circRNAs—circular RNA(s); BBB—blood–brain barrier; miRNA—microRNA; qPCR—quantitative polymerase chain reaction.

Biomarker	Citation	Sample Size (n)	Assay/Method	Sampling Timepoints	Main Finding/Effect Estimate
Netrin-1	Guo et al. 2019 [49]	638 AIS patients	ELISA (serum)	Within 24 h of admission	Higher serum Netrin-1 associated with lower risk of death or major disability at 3 months (adjusted OR ≈ 0.42, 95% CI 0.25–0.71).
	Zang et al. 2021 [50]	438 AIS patients	ELISA (serum)	<48 h after onset	Elevated Netrin-1 correlated with a favorable functional outcome (mRS ≤ 2); serum lipid levels modulated the effect.
	Luo et al. 2022 [51]	—(review + experimental)	Literature synthesis	—	Netrin-1 is implicated in neuroprotection, angiogenesis, and inflammation modulation post-stroke.
Omentin-1	Xu et al. 2018 [52]	150 AIS patients	ELISA (serum)	Within 24 h of admission	Higher baseline omentin-1 independently predicted good 3-month outcome (adjusted OR 0.38, 95% CI 0.17–0.82).
	Lin et al. 2021 [53]	210 AIS patients + 100 controls	ELISA	Within 72 h of stroke onset	Serum omentin-1 inversely correlated with carotid IMT and CRP; higher levels were associated with reduced stroke severity.
	Souza-Batista et al. 2007 [54]	100 obese vs. non-obese adults	ELISA/mRNA expression	Baseline (non-stroke)	Demonstrated omentin-1′s metabolic and anti-inflammatory function—relevant to vascular protection.
Circular RNAs	Zhang et al. 2023 [55]	Pooled n = 1254 (8 studies)	qRT-PCR	Acute phase (≤7 days)	Elevated circulating circRNAs (e.g., circFUNDC1, circPDS5B, circCDC14A) are associated with poor functional outcome and larger infarct volume (pooled OR ≈ 2.1).
	Zeraatiannejad et al. 2023 [56]	110 AIS patients + 110 controls	qRT-PCR	Within 72 h of onset	circDLGAP4 and circ_0008980 were elevated in AIS; higher levels are predicted to predict poor 3-month mRS.
	Siracusa et al. 2023 [57]	—	Literature synthesis	—	Summarized circRNA regulatory roles (oxidative stress, BBB integrity, apoptosis) in experimental stroke models.
MicroRNAs/ncRNAs	Mainali et al. 2025 [58]	204 AIS patients	RNA sequencing + validation by qPCR	≤24 h post-stroke	Identified distinct miRNA profiles predictive of functional outcome and inflammatory status post-AIS.

**Table 6:** Evidence summary for imaging and electrophysiologic biomarkers [86,87,88,89,90,91,92,93,94,95].

Modality	Key Metric	Temporal Sensitivity	Clinical Utility
DTI	CST lesion load, FA asymmetry	Hyperacute → chronic	Predicts motor outcome; complements clinical scales
fMRI	Resting-state connectivity, task-based activation	Acute → subacute → chronic	Tracks network reorganization; guides neuromodulation
EEG	Theta, alpha, beta power/coherence	Subacute → chronic	Bedside assessment; predicts therapy response; BCI applications
TMS	MEP presence/absence, cortical excitability	1–2 weeks post-stroke	Early upper limb prognostication (PREP2); guides rTMS therapy

## Data Availability

Not applicable.

## Abbreviations

The following abbreviations are used in this manuscript:

AIS	Acute Ischemic Stroke
ASK1	Apoptosis Signal-Regulating Kinase 1
BMI	Body Mass Index
circRNAs	Circular Rnas
CNNs	Convolutional Neural Networks
CSF	Cerebrospinal Fluid
DBIL	Direct Bilirubin
DTI	Diffusion Tensor Imaging
EEG	Electroencephalography
END	Early Neurological Deterioration
EVT	Endovascular Thrombectomy
FA	Fractional Anisotropy
fMRI	Functional Network Dynamics
IBIL	Indirect Bilirubin
LSTM	Long Short-Term Memory
MEPs	Motor Evoked Potentials
mRS	Modified Rankin Scale
ncRNAs	Noncoding Rnas
NfL	Neurofilament Light Chain
NIHSS	National Institutes Of Health Stroke Scale
RNNs	Recurrent Neural Networks
ROS	Reactive Oxygen Species
TBIL	Total Bilirubin
TMS	Transcranial Magnetic Stimulation
Trx	Thioredoxin
